# Book Review

**Published:** 1939

**Authors:** 


### Reviews of Books

**Treatment of Some Common Diseases.** Edited by T. Rowland Hill, M.D., M.R.C.P. Pp. xvi., 398. Illustrated. Edinburgh: E. & S. Livingstone. 1939. Price 15s.—This is a happily conceived symposium by the staff of the Southend-on-Sea Hospital. Dr. Rowland T. Hill defines the aims of his coadjutors. The volume they present is lucid and well illustrated. Among the contents are cerebro-vascular disease, anæmia, broncho-pulmonary suppuration, head injuries and X-ray therapy, immunization and anæsthesia. Children and their digestive disorders are tenderly handled: a relevant study is that of dental caries. Under hæmorrhage from the intestinal tract will be found the argument for a liberal diet in peptic ulcer (Dr. R. H. Campbell) now in current controversy. Uterine hæmorrhage is considered in the light of the endocrines as well as of surgical relief. The accoucheur, harassed in his lawful occasions, will appraise the insight and judgement of the article on delay in labour (Dr. Aleck W. Bourne). Mr. Wilfred Trotter describes his methods in malignant disease of the pharynx, and prostatic enlargement, that manifold infirmity of age, comes under survey by Sir I. de C. Wheeler.

**The Heart-Sounds in Normal and Pathological Conditions.** By O. Orias, M.D., and E. Braun-Menendez, M.D. Pp. xx., 258. Illustrated. London: Oxford University Press (Humphrey Milford). 1939. Price 15s.—This is a book for the specialist, but it presents a very readable account of the heart-sounds and their causes. It is based largely on numerous graphic records obtained by the authors and their colleagues, together with their own interpretations. After preliminary chapters on the principles and technique of phonocardiography comes a large section on the normal heart-sounds, all of which are considered in detail; this is followed by chapters on the sounds in pathological conditions. One gets a very satisfactory impression of the discussions in these sections. The most noticeable feature of the book is the wealth of phonocardiograms, mostly with simultaneous venous pulse records, electrocardiograms, etc. ; as many of these have appeared previously in Argentine journals there is ample justification for their collection and publication in one book in England. The records are well produced, though the reproductions of portraits at the beginning are disappointing. There is a comprehensive list of references and an author index, and the subject index is good. The format and print leave nothing to be desired.

**Physiotherapy in Medical Practice.** By H. MORRIS, M.D., D.M.R.E. Pp. viii., 276. Illustrated. London : Edward Arnold & Co. 1939. Price 12s. 6d.—The title of Dr. Hugh Morris's latest volume is somewhat misleading, as he only writes on medical electricity and " light." The term " physiotherapy " covers a far wider field. On the whole the contents of the book are sound, well-presented, and can be thoroughly recommended to physicians who are interested in electricity and " light." There are some criticisms, however. The short chapter on Physical Principles is perhaps adequate enough, but we wonder why so much space is given to explaining the terms series and parallel, which surely everyone understands ; yet we look in vain for any explanation as to how the metal rectifier works. Why is it usual in text-books of this sort to give so much space to photographs of the outsides of machines, without giving working diagrams as well ? Do such illustrations as Figs. 21, 47, 48, 74, 75, 77 and 88, to mention only a few, help at all ? It is a pity that burns are not taken into account more, both as regards the cause and treatment. " . . . scalds may be produced by evaporation of sweat " is somewhat misleading. The diagrams, as a whole, are excellent, but Fig. 91, illustrating the angle of incidence, is not quite clear, especially as there is a misprint in the explanation ! There is no bibliography, but there is an appendix giving the Government Memorandum on Electro-Medical Apparatus, and there is also a glossary. In the index, the principal references are given in bolder figures than the rest : this should be universal in text-books.

**A Survey of Child Psychiatry.** Edited by R. G. GORDON, M.D., D.Sc., F.R.C.P. Pp. xii., 278. London : Oxford University Press (Humphrey Milford). 1939. Price 10s. 6d.—This volume, issued by the British Child Guidance Council, deals with conditions applicable to this country. It is composed of twenty-two articles by twenty-one physicians, and the editor has taken care that repetitions do not occur. The book deals with the psychological aspects of physical and mental conditions, with sociology and with special syndromes. Although the majority of the articles are for the doctor, some are elementary and more suitable for the student. The typography is clear, but the practice of using small print for case details is trying. The indexing is good, the general index consisting of nine pages. Unfortunately, some of the references given are not complete and accurate. The book is sound and will be most useful to those taking up child psychiatry, and to others who deal with psychological difficulties in children.

**Pulmonary Tuberculosis.** By G. G. KAYNE, M.D., M.R.C.P., D.P.H., W. PAGEL, M.D., and L. O'SHAUGHNESSY, M.D., F.R.C.S. Pp. xvi., 565. Illustrated. London: Oxford University Press (Humphrey Milford). 1939. Price 42s.—This important work cannot be overlooked by members of the profession seriously interested in the control and treatment of pulmonary tuberculosis. The authors are well known for their contributions to the literature of tuberculosis, and this particular combination of physician, pathologist and surgeon has ensured that to each of the divergent aspects of this vast subject is brought an intimate and extensive knowledge. The general editorship of Dr. Gregory Kayne has allowed the influence of each collaborator to be felt in all its sections. The result is a book of the greatest interest to the physician or surgeon dealing with pulmonary tuberculosis, and the whole field, apart from the details of clinical examination, is ably covered. The hope expressed by the editor that the medical student will obtain from it a sense of proportion is probably doomed to disappointment. It is essentially a book for the specialist, who will find the numerous problems which beset him discussed in a constructive spirit which is not always evident in books dealing with this subject. The bibliography at the end of each chapter is very extensive, and will be appreciated by the advanced worker. As an example of the profusion of references may be cited the short chapter of four and a half pages devoted to a historical review of collapse therapy. This section has a bibliography of nearly four pages, with about one hundred references. The illustrations are very numerous and are beautifully reproduced. The skiagrams reproduced are admirable both in their selection and quality, and it is only rarely that too great a profusion of pointing arrows seems to cast some doubt on the reader's intelligence.

An illustration of anthracosis would be welcome, as it is stated that the radiological appearances are similar to those of silicosis, which is not the commonly accepted view. Some of the recommendations under diagnosis and control seem Utopian. For example, when the diagnosis of cancer of the lung is discussed, it is baldly stated that in cases of hæmoptysis "when the patient is over forty he should always be bronchoscoped." Tubercle bacilli in the sputum apparently justify no exception. The chapters on treatment provide a thoroughly up-to-date discussion of the possibilities for all types of patients, and there must be few specialists who would not gain from their perusal. This book is very highly recommended.

**Royal Northern Operative Surgery.** By THE SURGICAL STAFF OF THE ROYAL NORTHERN HOSPITAL ; edited by SIR L. E. BARRINGTON-WARD, K.C.V.O., F.R.C.S. Pp. x., 551. Illustrated. London : H. K. Lewis & Co. Ltd. 1939. Price 42s.—Surgeons have had a reputation in certain quarters in the past for jealousy of their colleagues, and factiousness ; it is good to see that ten of them on one hospital staff can collaborate to write a book, and an excellent book, too. It is fresh and up to date, well illustrated, and includes a description of nearly all the operations a surgeon is likely to perform, in less than 600 pages of large, clear print. It would take too much space to go through the whole volume selecting interesting paragraphs here and there for notice. It must suffice to say that the choice of methods shows good judgement, and inferior or obsolete techniques are not described. The book is wide in scope, including even gynæcology. By the way, why is it that so few textbooks of operative surgery, this being no exception, describe a method such as Kronlein's for approaching growths of the orbit ? To sum up, this is a thoroughly useful book, which every surgeon should possess.

**Cæsarean Section—Lower Segment Operation.** By C. M. MARSHALL, F.R.C.S. Pp. viii., 230. Illustrated. Bristol : John Wright & Sons Ltd. 1939. Price 21s.—It is only in comparatively recent years that the lower segment Cæsarean operation has been widely used in this country. Its importance now is everywhere realized and acknowledged. There is no doubt that the operation is a specialized one, and we strongly recommend every operating obstetrician to read this book. The author surveys the history and technique of the operation in considerable detail, stating his own preferences and reasons. Every possible difficulty is dealt with and discussed in full.

The illustrations are admirable, and Messrs. John Wright have maintained their usual high standard of printing and lay-out. It is not too much to say that this book should find a place on the bookshelf of every operating obstetrician. Mr. McIntosh Marshall is to be congratulated on having produced a book which deals with the operation so thoroughly in all its aspects.

**Urine Examination and Clinical Interpretation.** By C. E. DUKES, M.Sc., M.D. Pp. xvi., 403. Illustrated. London: Oxford University Press (Humphrey Milford). 1939. Price 25s.—This monograph, which contains practically everything there is to know about urine and its examination, should find its way to the shelf of every pathologist and also of anyone interested in diseases of the urinary tract. Two of its outstanding characteristics are the very clear, concise clinical interpretations of the results of each test and the brief but experienced criticism of each method. Simple methods are preferred where possible, and the general practitioner will here find the tests of renal function well explained and described in order of simplicity and applicability. Microscopical examination of the urine is dealt with fully, including pictures of substances and foreign bodies which can so easily be mistaken for casts and other deposits of pathological significance. The estimation of output of vitamins and hormones is included, and there is also a good chapter on the examination of urine for drugs in cases of poisoning. In these days of biochemistry and clinical pathology, the examination of the urine has been rather thrust in the background, while that of the blood has taken pride of place. This book reveals the wealth of knowledge which can be gained from the examination of the urine, and will do much to reinstate its former importance. It contains many fine illustrations and is set out in easily-read large print.

**The March of Medicine.** By R. L. WILBUR, M.D. Pp. x., 280. London: Oxford University Press (Humphrey Milford). 1938. Price 12s. 6d.—This volume consists of various addresses and articles on Medical Topics composed by Dr. Wilbur, President of Stanford University, between the years 1913–37. As might be expected, medical education forms the groundwork of most of the articles. Some of them are prophetic, some are platitudinous, but few have any constructive ideas. They might furnish the sort of material that a Minister of Health might find useful in composing a public oration. The addresses may have sounded fine when they were delivered, they may have been appropriate to the occasion, but the title of the collection "The March of

Medicine " seems to hold out promise of something like a survey of progress. Actually the " March " when read is terribly like the tramp of men marking time. No, on second thoughts, it is just a cluster of speeches made by any distinguished personage at any school prize-giving.

**Claude Bernard—Physiologist.** By J. M. D. OLMSTED, M.A., Ph.D. Pp. 318. Illustrated. London : Cassell & Co. Ltd. 1939. Price 15s.—Professor Olmsted has written a delightful biography of Claude Bernard, bringing out not only the importance of his physiological discoveries, but also a great deal of new information about his life and personal contacts. Just forty years ago the late Sir Michael Foster wrote an account of Bernard in the " Masters of Medicine " series, published by Fisher Unwin, but although it brought to our knowledge the surpassing contributions Bernard made to physiology, it gave very little idea of the man himself. Now, in Olmsted's life of Bernard, we can follow the details that went to make up the man, we see his human side and a very human side it was. We read of his ill-starred marriage and of his long continued ill-health. We find him a poor lecturer, a not very convincing teacher, a scientist who made plenty of mistakes over his discoveries, and yet remains one of the greatest physiologists who ever lived. For, as Professor Olmsted says in his preface, "the structure of physiology rests on sure foundations which he laid." Physiology, whilst owing much to his magnificent discoveries, owes more, far more, to the experimental method of inquiry which he so inimitably expounded. This is not a book to be dipped into for quotable gems, it is a book to be read through from cover to cover.

**Human Gastric Secretion.** By B. J. E. IHRE, M.D. Pp. 232. London : Oxford University Press (Humphrey Milford). 1939. Price 12s. 6d.—This work is really a long scientific paper, reprinted in book form, describing observations on normal and pathological gastric secretion and discussing the results. Gastric juice was obtained by continuous aspiration through a Lagerlöf-Ågren combined gastric and duodenal tube following stimulation by histamine and insulin hypoglycæmia. The pathological cases included subjects of gastric ulcer, duodenal ulcer, chronic gastritis and pernicious anæmia. There is an excellent chapter on the secretion of acid and acidity regulation in which Teorell's hypothesis of acidity regulation by diffusion receives support. The author has found no evidence of hyperacidity as distinct from hypersecretion. The volume, which is well arranged and copiously illustrated with case records, secretion curves, etc., suitably grouped together at the end, is a publication for the specialist and is not likely to appeal to the general practitioner ; the type and occasional spelling inaccuracies are evidence of foreign printing, but do not detract from its value.

**Sanitary Law in Question and Answer.** Fourth Edition. By C. PORTER, M.D., B.Sc., M.R.C.P., and J. FENTON, C.B.E., M.D., M.R.C.P., D.P.H. Pp. xvi., 352. Illustrated. London : H. K. Lewis & Co. Ltd. 1939. Price 10s.—The fact that this book has attained its fourth edition proves that it is meeting a demand. The first edition was published in 1910 and, as is the case with all Public Health books, owing to rapid changes in Acts of Parliament and the views of Public Health authorities, this last edition is entirely different from the first. For those who wish to study Public Health in the form of question and answer this book is admirably suited. It covers the course on Public Health given to most medical students studying for their final qualifying examination.

**Sanitary Inspector's Handbook.** Fourth Edition. By H. H. CLAY, F.R.San.I., F.I.S.E. Pp. xxii., 528. Illustrated. London : H. K. Lewis & Co. Ltd. 1939. Price 17s. 6d.—The fourth edition of this useful and comprehensive handbook appears with the coming into operation of the Food and Drugs Act, 1938, consequently the chapters dealing with food control and meat inspection have been rewritten and brought up to date to conform with the new legislation. Advantage has been taken to revise and amplify other chapters, much new matter has been introduced in the light of later knowledge and further illustrations have been added. The arrangement is excellent, being divided into thirty-one chapters, each dealing with a specific aspect of the many diverse duties now laid upon a sanitary inspector with the appropriate legislation succinctly summarized in a very readable manner. The illustrations are finely drawn and most explicit in detail, and being furnished with a very full index makes the work a valuable text-book for use in all public health authorities. It can be recommended not only to the student, but also to the practical administrator. It was unnecessary to include on page 310 the provisions regarding bakehouses in the Factory and Workshops' Act, 1901, and included as the Third Schedule of the Factories Act, 1937, as these have since been repealed by the Food and Drugs Act, 1938.

